# Editorial: Advancements in diversity and drug resistance mechanisms in mycobacterial diseases

**DOI:** 10.3389/fmicb.2026.1812182

**Published:** 2026-03-02

**Authors:** Samira Tarashi, Manoj Baranwal, Svetlana Khaiboullina, Majid Marjani

**Affiliations:** 1 Department of Mycobacteriology and Pulmonary Research, Pasteur Institute of Iran, Tehran, Iran; 2 Microbiology Research Center (MRC), Pasteur Institute of Iran, Tehran, Iran; 3 Department of Biotechnology, Thapar Institute of Engineering and Technology, Patiala, India; 4 Department of Microbiology and Immunology, University of Nevada, Reno, NV, United States; 5 Clinical Tuberculosis and Epidemiology Research Center, National Research Institute of Tuberculosis and Lung Diseases (NRITLD), Shahid Beheshti University of Medical Sciences, Tehran, Iran

**Keywords:** antimicrobial resistance, diagnostics, drug resistance, genomics, *Mycobacterium tuberculosis*, non-tuberculous mycobacteria, therapeutics

The global tuberculosis (TB) epidemic is evidence of one of humanity's most enduring and adaptable microbial adversaries. In 2024 alone, the disease claimed an estimated 1.23 million lives, a stark statistic for a condition that is both preventable and curable (World Health Organization, 2025). The COVID-19 pandemic further strained diagnostic and treatment services, reversing hard-won progress and highlighting the fragility of our healthcare defenses (Williams, 2024). This persistence is fueled by the insidious rise of drug-resistant TB (DR-TB), declared a complex public health crisis by the WHO (Dheda et al., 2024; Ferdosnejad et al., 2024). Confronting DR-TB and the expanding threat of non-tuberculous mycobacterial (NTM) diseases demands a multi-pronged scientific strategy: precise surveillance, rapid diagnostics, a thorough understanding of their evolving defenses, and novel strategies to overcome them.

The present Research Topic presents a collection of 19 articles (17 original research articles, one review, and one methodology article) that collectively address these challenges. The studies presented here range from macro-scale epidemiology to molecular mechanisms, encompassing the diversity of the *Mycobacterium tuberculosis* complex and NTM pathogens. They provide not only a snapshot of the current battlefield but also critical tools and insights for the campaigns ahead.

First, effective action against a resistance epidemic depends on accurate intelligence to map its landscape. The monumental study by Xu et al. provides an essential update on the DR-TB profile in China by analyzing an unprecedented 55,388 strains over two decades. Their findings confirm a serious and complex epidemic, with high resistance rates to first-line drugs and the identification of 96 novel mutations. This work underscores the fact that resistance is not static but rather evolves, necessitating continuous genomic surveillance to keep diagnostic tools and treatment guidelines relevant. This national perspective is powerfully complemented by studies that drill down into local dynamics. For example, research by Huang et al. in Nanning and Wang D.-M. et al. on pediatric TB in southwest China provides local insights. These studies advance from description to prediction and prioritization by identifying high-risk patient groups, such as retreatment cases, diabetic individuals, and adolescent girls in specific ethnic communities, enabling targeted public health interventions in areas where resources are needed the most. These studies highlight that the epidemic's manifestation varies locally, necessitating tailored public health responses that address specific demographic and geographic vulnerabilities.

In addition, deciphering the molecular mechanisms of resistance is fundamental to understanding how mycobacteria evade treatment. Several articles in this Research Topic decode this phenomenon at the molecular level. Research from Singh et al. in India and Alvarado-Peña et al. in Mexico uses molecular diagnostics and whole-genome sequencing to catalog the mutational landscape of DR-TB, confirming global patterns while uncovering region-specific variants and underscoring the potential role of efflux pumps. At a more fundamental level, Li M. et al. delve into the more subtle concept of drug tolerance, investigating a novel regulatory pathway involving the *Rv0274* gene that influences isoniazid susceptibility. This work sheds light on the complex genetic networks that bacteria use to survive antibiotic stress, which may precede the acquisition of high-level resistance. Technological advancements support this mechanistic understanding. Li L. et al. present a breakthrough CRISPR/Cas9-based tool for *Mycobacterium abscessus*, finally enabling efficient genetic manipulation of this notoriously difficult-to-treat NTM. This will accelerate the functional validation of resistance and virulence genes identified in genomic studies, such as Wang J. et al.'s study of the *M. abscessus* complex in Hainan.

Translating this knowledge into an impact on patients requires innovation at the point of care and in treatment regimens. In terms of diagnostics, Ou et al. evaluate a new automated molecular test (GenMax) for the simultaneous detection of TB and resistance to rifampicin and isoniazid. While its sensitivity requires further optimization to meet WHO targets, such integrated, rapid platforms are crucial for decentralized testing and managing DR-TB. Furthermore, Tamblin et al. address a persistent technical hurdle by refining pyrazinamide susceptibility testing at neutral pH, offering a more reliable method to guide the use of this key drug. The therapeutic pipeline is also being replenished. Davids et al. demonstrate the promising antimycobacterial activity of the engineered peptide NZ2114, which shows synergy with first-line drugs and efficacy in an animal model. Ilchenko et al. explore the repurposing of gallium citrate, demonstrating that its combination with levofloxacin disrupts metabolic pathways in MDR-TB and enhances bacterial killing, revealing a novel combinatorial strategy.

The challenge extends beyond TB, as highlighted by work on NTM. Studies by Wang Y. et al. on *M. kansasii* and by Fernandez-Pittol et al. on slow-growing NTM provide crucial drug susceptibility data, validating the efficacy of newer TB drugs, such as bedaquiline, against these pathogens and reinforcing the need for culture-guided, personalized therapy. Finally, broadening our perspective, the review by Zhang et al. on *Candida auris* serves as a crucial parallel. It reminds us that the challenges of rapid spread, environmental persistence, and pan-drug resistance are not unique to mycobacteria. The infection control strategies and diagnostic imperatives discussed are highly relevant, emphasizing that the fight against antimicrobial resistance is a unified field.

In conclusion, the collective work presented in this Research Topic vividly illustrates that combating mycobacterial diseases is a multi-dimensional challenge. Success depends on integrating intelligence across all scales, from global and national surveillance networks to the prediction of individual risk, from cataloging common resistance mutations to elucidating fundamental survival pathways, and from improving existing diagnostics to deploying next-generation genetic tools and therapeutic agents.

The diversity of mycobacterial species and their resistance mechanisms is vast, as shown by studies on different lineages of *M. tuberculosis* (Qiu et al., Liu et al.), *M. marinum* pangenomics (Shahed et al.), and environmental NTM detection (Ferraro et al.). Confronting this diversity requires an equally diverse and agile toolkit. The research compiled here provides essential new components for that toolkit (including data, methods, and molecules) that can empower clinicians, public health officials, and scientists to design more precise, effective, and timely interventions. As we move forward, the integration of these advances into policy and practice will be critical to reducing the unacceptable mortality of TB and other mycobacterial diseases, steering us toward the goal of a world free from their burden.
